# Risk of injuries before and after a diagnosis of cirrhosis: A population-based cohort study

**DOI:** 10.1097/HC9.0000000000000238

**Published:** 2023-10-12

**Authors:** Ying Shang, Qing Shen, Elliot B. Tapper, Axel Wester, Hannes Hagström

**Affiliations:** 1 Department of Medicine, Huddinge, Karolinska Institutet, Stockholm, Sweden; 2 Center of Public Health Sciences, University of Iceland, Reykjavík, Iceland; 3 Unit of Integrative Epidemiology, Institute of Environmental Medicine, Karolinska Institutet, Stockholm, Sweden; 4 Department of Internal Medicine, Division of Gastroenterology and Hepatology, University of Michigan, Ann Arbor, Michigan, USA; 5 Unit of Hepatology, Department of Upper GI Diseases, Karolinska University Hospital, Stockholm, Sweden

## Abstract

**Background:**

Cirrhosis is often asymptomatic prior to decompensation. Still, cognitive impairment and sarcopenia may be present before decompensation, possibly increasing the risk of injuries. We estimated the risk of injuries during the period shortly before and after cirrhosis diagnosis.

**Methods:**

All patients (N=59,329) with a diagnosis of cirrhosis from 1997 to 2019 were identified from the Swedish National Patient Register. We used a self-controlled case series design to compare the incidence rates (IR) of injuries during a “diagnostic period” (within 3 months before or after the cirrhosis diagnosis date) to a self-controlled “prediagnostic period” (the same 6 calendar months 3 years before diagnosis), using conditional Poisson regression. Injuries were ascertained from the National Patient Register.

**Results:**

We identified 23,733 (40.0%) patients with compensated and 35,595 (60.0%) with decompensated cirrhosis. There were 975 injuries (IR 2.8/1000 person-months) during the prediagnostic period, and 3610 injuries (IR 11.6/1000 person-months) identified during the diagnostic period. The IR ratio was 8.1 (95% CI 7.5–8.7) comparing the diagnostic period with the prediagnostic period. For patients with compensated cirrhosis, the risk increment of injuries was highest just before the diagnosis of cirrhosis, whereas the risk increase was highest shortly after the diagnosis for those with decompensation.

**Conclusions:**

The incidence of injuries increases shortly before and after the diagnosis of cirrhosis. These findings indicate that cirrhosis is frequently diagnosed in conjunction with an injury, and highlight the need for injury prevention after cirrhosis diagnosis, especially in patients with decompensation.

## INTRODUCTION

Liver cirrhosis is an increasing cause of mortality worldwide,^1^ and is the second cause of years of working life lost in Europe.^2^ Compensated cirrhosis is asymptomatic and thus often underdiagnosed until decompensation occurs. Cirrhosis is a catabolic state in which muscle protein breakdown exceeds synthesis resulting in sarcopenia, and osteoporosis is common. Sarcopenia, hepatic encephalopathy, and osteoporosis all increase the risk of falls and fractures.^3–5^ Other injury type, such as self-harm, triggered by psychological stress and depression, has also been linked to decompensated cirrhosis.^6,7^

Studies examining the risk of injuries were restricted to either the time after cirrhosis diagnosis,^8–10^ or after liver transplantation.^11^ It is possible that these underlying sarcopenia and cognitive impairment, may precede the formal diagnosis of cirrhosis,^12,13^ which might increase the risk of injuries even before cirrhosis diagnosis. However, evidence on the risk of injuries during a formal cirrhosis diagnosis is sparse.

The pattern of injuries might vary across etiologies and severity of cirrhosis. An increased risk of fractures was shown in patients with alcohol-associated cirrhosis,^9,10^ while the findings for patients with cirrhosis due to viral hepatitis are contradictory.^14,15^ Various study populations with mixed cirrhosis etiologies and adjustments for different confounding factors make it difficult to directly compare results across studies. Therefore, we assessed the risk of injuries during cirrhosis diagnosis and examined the patterns of injuries by etiology and severity of cirrhosis using Swedish nationwide registers.

## METHODS

### Study population

We used the data from the ongoing Decoding the Epidemiology of Liver Disease in Sweden cohort, which contains administrative data on all patients with a diagnosis of liver disease from the National Patient Register (NPR) and other registers since 1964.^16^ The NPR compiles hospital inpatient records from 1964 onward and is nationwide since 1987. Further, the NPR covers specialized outpatient visits since 2001, but does not include primary care.^17^ For each patient with liver disease, up to 10 individuals from the general population (controls) were randomly selected and matched on age at diagnosis, sex, and municipality.^18^

In this study, we identified patients with any diagnosis of cirrhosis from inpatient care since 1997 and outpatient visits since 2001 until 2019, based on ICD-10 codes.^17^ We classified cirrhosis as compensated or decompensated, with compensated cirrhosis further classified into alcohol-associated cirrhosis or cirrhosis due to other etiologies. Patients with decompensated cirrhosis were not subclassified based on etiology, since coding for underlying etiology was only present in 42.7% of the cases. This was not an issue for compensated cirrhosis, where a specific ICD-10 code exists for alcohol-associated cirrhosis. Since there is no specific ICD-10 code for HE, patients with HE were identified using an algorithm based on either having a treatment for HE (ie, rifaximin and/or lactulose), combined with an ICD-code for compensated cirrhosis.^17,19^ The treatment information was obtained from the Swedish Prescribed Drug Register, which was initiated since July 2005.^16^ The PPVs of ICD-10 codes for cirrhosis are 95% for alcohol-associated cirrhosis, 91% for unspecified cirrhosis, and 96% for esophageal varices, according to a previous validation study.^20^ However, this study reported the low PPV (43%) of using the standalone code of ascites to identify cirrhosis. We, therefore, defined ascites as hepatic, using the codes of ascites in combination with not having a code of heart failure or nonliver cancers within 1 year before ascites (Supplemental Table S1 for definitions, http://links.lww.com/HC9/A561).

### Study design

We used the self-controlled case series method to investigate the association between cirrhosis and injuries.^21^ This method uses data only on individuals with cirrhosis and compares the incidence of injuries during periods in which the individual is considered to be at the higher risk of injuries (in this case, during a diagnosis of cirrhosis), to the incidence during all other periods. Therefore, this method begs the question of “when” risk is increased, instead of “who” is at risk. Using this method, time-invariant confounders (eg, genetic factors or prevalent chronic comorbidities) are automatically adjusted for, as individuals act as their own controls.

**FIGURE 1:**
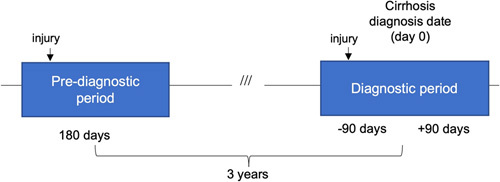
Overview of the study design.

The follow-up time for each patient with cirrhosis was divided into 2 periods: a diagnostic period in conjunction with the diagnosis of cirrhosis, defined as the risk period, and a prediagnostic period, defined as the reference period (Figure 1). We defined the date of cirrhosis diagnosis as day 0. Because the maximum waiting time for referral to the first specialist visit is 90 days according to the Swedish statutory health care regulations,^22^ we defined the risk period (“diagnostic period”) as 90 days before until 90 days after the date of cirrhosis diagnosis (day 0), or 6 months in total. Accordingly, we defined the same 6 months 3 years before the diagnosis date as the reference period (“prediagnostic period”). We chose 3 years as the time interval because the incidence rate (IR) of injuries started to increase around 3 years before the diagnosis of cirrhosis, after comparing the incidence of injuries in patients to that of matched controls from the general population of the same age (Supplemental Table S2, http://links.lww.com/HC9/A561 and Supplemental Figure S1, http://links.lww.com/HC9/A561).

### Injury ascertainment

Among patients with cirrhosis, we ascertained the first health care contact due to injuries from both inpatient and outpatient contacts with health care recorded in the NPR during each of the prediagnostic and diagnostic periods, respectively, using ICD-9 and ICD-10. The PPV for identifying injuries in the NPR is 94.8%.^17^ We studied the first injury as the outcome rather than the total number of injuries during each period because consecutive injuries within a short period may be dependent on previous injury events (eg, coding for a new fracture at the same site might represent a new fracture but also a repeated visit for the first fracture).

Injuries were classified as falls, fractures, or self-harm. Fractures were categorized by location (skull/vertebrae/ribs/sternum, hip/pelvis, extremities), and by mechanism (osteoporotic fractures, high-energy fractures, other fractures with nonosteoporotic nor high-energy related) (definitions in Supplemental Table S1, http://links.lww.com/HC9/A561). Self-harm was defined as coding representative of deliberate self-inflicted poisoning or injury and did not include assault by another person with intent to injury or accident.

### Covariates

Information on years of formal education and cohabitating status was retrieved from the 1997 Swedish population and housing census. Pre-existing comorbidities (including diabetes, psychological disorders, dementia, cardiovascular diseases, osteoporosis, and HCC) were identified from the NPR (Supplemental Table S1, http://links.lww.com/HC9/A561).

### Statistical analysis

We calculated IRs (per 1000 person-months) of injuries during the prediagnostic and diagnostic periods by dividing the number of injuries over the sum of person-months at risk during each period. The incidence rate ratio (IRR) and 95% CI were estimated by fitting a conditional Poisson regression model, which compares the IR of injuries during the diagnostic period to the IR during the prediagnostic period for the same individuals rather than across groups, thus accounting for time-invariant confounders.^23^ We calculated and plotted IRRs from day −90 to −60, −59 to −30, −29 to −1, 0–29, 30–59, and 60–90 in the diagnostic period compared to the respective 6 periods from the prediagnostic period. By design, every patient must survive to the diagnostic date. For the patients who died or emigrated 90 days after cirrhosis diagnosis, we censored patients at the corresponding date. IRRs were calculated by clinically important subgroups such as categories of age, sex, and pre-existing comorbidities. We also investigated the risk of the injury subtypes in patients with cirrhosis.

Subgroups analyses were performed for different cirrhosis severity and etiologies. Sensitivity analyses are provided in the Supplemental Figure S1, http://links.lww.com/HC9/A561 and Supplemental Table S3, http://links.lww.com/HC9/A561, which includes (1) comparison of IRs of injuries preceding the cirrhosis diagnosis for patients and matched individuals to assess the impact of age on the occurrence of injuries, and (2) analysis confined to patients who survived the diagnostic period to determine whether censoring at death would affect the associations in a meaningful way. The assumption of equal dispersion in Poisson regression was found to hold for all analyses. All statistical analyses were performed with Stata version 17 (StataCorp LP).

### Ethical considerations

The study was approved by the Regional Ethics Review Board in Stockholm (dnr 2017/1019-31/1). Since this study included analyses of deidentified data, written consent from participants was not required.

### Patient and public involvement

No patients or members of the public were involved in the development of research questions, the design of the study, interpretation, or writing the results. We plan to disseminate the results of our research to the relevant patient community.

## RESULTS

### Patients characteristics

We identified 59,329 patients with a diagnosis of cirrhosis from 1997 to 2019 (Table 1), of which 21,717 (89%) were first identified from the outpatient visits and 2563 (11%) were from the inpatient care. Among all patients with cirrhosis, 23,733 (40.0%) had compensated cirrhosis and 35,595 (60.0%) with decompensated cirrhosis.

**TABLE 1:** Characteristics of patients with cirrhosis (n=59,329)

Characteristics	No. (%)/median (IQR)
Age at diagnosis, y	62 (50–71)
Female	25,311 (43)
Education, y	
≤9	22,144 (37.3)
>9	35,250 (59.4)
Unknown	1,935 (3.3)
Cohabitation status	
Yes	31,890 (53.8)
No	10,137 (17.1)
Unknown	17,302 (29.2)
Calendar year of diagnosis	
1997–2000	6971 (11.7)
2001–2010	20,861 (35.2)
2011–2019	31,497 (53.1)
Diabetes	2762 (4.6)
Psychological disorder	13,460 (22.7)
Cardiovascular disease	82,862 (14.0)
Dementia	222 (0.4)
Osteoporosis	342 (0.6)
Compensated cirrhosis	23,733 (40.0)
Alcohol-associated cirrhosis	12,084 (20.4)
Cirrhosis due to other etiologies	11,649 (19.6)
Decompensated cirrhosis	35,595 (60.0)
Ascites	29,821 (50.3)
Bleeding esophageal varices	5393 (9.1)

The median age at cirrhosis diagnosis was 62 (interquartile range: 50–71) years, and 25,311 (43%) were female. More than half of the patients had education over 9 years (59.4%) or were cohabiting (53.8%). A total of 2767 patients (4.6%) were diagnosed with diabetes, 13,460 (22.7%) had any previous psychological disorder, and 222 (0.4%) had dementia. Cardiovascular diseases and HCC were found in 8282 (14.0%) and 97 (0.2%) patients, respectively, at or before cirrhosis diagnosis.

### Association between cirrhosis and injuries

During the diagnostic period, we identified 3610 injuries (IR 11.6/1000 person-months) compared to 975 in the prediagnostic period (IR 2.8/1000 person-months), translating to an IRR of 8.1 (95% CI 7.5–8.7) (Table 2). Among the injury cases, 300 (8.3%) and 77 (8.0%) were identified only from the inpatient care during the diagnostic period and prediagnostic period, respectively. In patients with compensated and decompensated cirrhosis, there were 473 (IR 3.4/1000 person-months) and 502 (IR 2.4/1000 person-months) injuries identified during the prediagnostic period, and 1933 (IR 15.1/1000 person-months) and 1677 (IR 9.0/1000 person-months) injuries during the diagnostic period, respectively. This translated to an IRR of 8.9 (95% CI 8.1–9.9) for the risk of injuries in compensated cirrhosis and an IRR of 7.0 (95% CI 6.1–7.7) in decompensation during the diagnostic period compared to the prediagnostic periods.

**TABLE 2:** Incidence rates (IR) per 1000 person-months and incidence rate ratios (IRR) of injuries during the diagnostic period compared to the prediagnostic period in 59,329 patients with cirrhosis, 1997–2019 in Sweden

	Prediagnostic period		Diagnostic period			
	No. injuries	IR	No. injuries	IR	IRR (95% CI)	*p*
Total sample						
All patients with cirrhosis	975	2.8	3,610	11.6	8.1 (7.5–8.7)	<0.001
Compensated cirrhosis	473	3.4	1,933	15.1	8.9 (8.1–9.9)	<0.001
Alcohol-associated cirrhosis	306	4.3	1,144	17.7	7.9 (6.9–9.0)	<0.001
Compensated cirrhosis due to other etiologies	167	2.5	789	12.6	11.0 (9.4–13.1)	<0.001
Decompensated cirrhosis	502	2.4	1,677	9.1	7.0 (6.1–7.7)	<0.001
Bleeding esophageal varices	67	2.6	224	9.6	7.3 (5.5–10.1)	<0.001
Ascites	374	2.2	1,288	8.6	7.1 (6.3–8.1)	<0.001
By demographics and comorbidities						
Age at cirrhosis diagnosis (y)						
≤55	411	3.2	1200	10.6	5.7 (5.1–6.5)	<0.001
56–65	225	2.7	800	10.6	7.6 (6.5–8.9)	<0.001
66–75	186	2.2	845	11.2	10.4 (8.7–12.3)	<0.001
≥76	153	2.7	765	15.9	12.2 (10.1–14.7)	<0.001
Sex						
Men	550	2.7	2141	11.9	8.7 (7.8–9.6)	<0.001
Women	425	2.8	1469	11.0	7.2 (6.4–8.1)	<0.001
Education (y)						
≤9	376	2.9	1502	13.1	8.7 (7.7–9.8)	<0.001
>9	572	2.7	1985	10.6	7.4 (6.7–8.3)	<0.001
Unknown	27	2.3	123	12.3	9.5 (6.1–14.7)	<0.001
Cohabitation status						
Yes	505	2.7	1835	10.7	7.8 (7.0–8.7)	<0.001
No	189	3.1	784	14.5	9.8 (8.2–11.8)	<0.001
Unknown	281	2.7	991	11.4	7.3 (6.3–8.4)	<0.001
Calendar year of cirrhosis diagnosis						
1997–2000	148	3.6	347	9.5	3.9 (3.2–4.9)	<0.001
2001–2010	320	2.6	1137	10.4	7.4 (6.4–8.4)	<0.001
2011–2019	507	2.7	2126	12.8	9.8 (8.8–10.9)	<0.001
Diabetes						
Yes	69	4.2	223	15.9	7.2 (5.3–9.7)	<0.001
No	906	2.7	3387	11.4	8.1 (7.5–8.7)	<0.001
Cardiovascular disease						
Yes	175	3.6	597	14.0	7.2 (5.9–8.6)	<0.001
No	800	2.6	3–013	11.1	8.2 (7.5–8.9)	<0.001
Psychological disorder						
Yes	456	5.8	1194	16.8	4.9 (4.4–5.5)	<0.001
No	519	1.9	2416	10.0	10.9 (9.8–12.1)	<0.001
HCC						
Yes	1	1.7	5	10.9	9.4 (1.1–83.1)	0.045
No	974	2.8	3605	11.6	8.0 (7.4–8.6)	<0.001
Dementia						
Yes	12	9.4	25	23.7	2.5 (1.2–5.4)	0.015
No	963	2.7	3,585	11.5	8.1 (7.5–8.7)	<0.001

We observed that older patients (aged ≥76 y) and those diagnosed at a later calendar period (2011–2019) had higher increases in the risk of injuries than younger patients (aged 65 y or below), and those diagnosed earlier, respectively (Table 2). Higher estimates of the relative risk of injuries were observed for those without pre-existing comorbidities, because these patients had much lower IRs of injuries during the prediagnostic period than those with these comorbidities.

Compared to the prediagnostic period, the incidence of injuries was high throughout the full diagnostic period. The incidence started to increase substantially 1 month before (IRR: 7.9 95% CI 6.7–9.3) and peaked 1 month after (IRR: 8.6, 95% CI 7.2–10.2) the diagnosis of cirrhosis (Table 3). The risk decreased rapidly thereafter but remained increased compared to the prediagnostic period.

**TABLE 3:** Number and incidence rate ratio (IRR) of injuries during the diagnostic period compared to the prediagnostic period

Risk period: days	No. injuries	IRR^a^ (95% CI)
All cirrhosis		
−90 to −60	400	3.5 (2.9–4.3)
−59 to −30	418	3.2 (2.6–3.8)
−29 to −1	1151	7.9 (6.7–9.3)
0–29	1026	8.6 (7.2–10.2)
30–59	354	3.2 (2.6–4.0)
60–90	261	2.8 (2.2–3.6)
By cirrhosis severity		
Compensated cirrhosis		
−90 to −60	204	4.1 (3.1–5.5)
−59 to −30	207	3.0 (2.3–3.8)
−29 to −1	912	10.4 (8.3–12.9)
0–29	362	7.4 (5.6–9.9)
30–59	129	3.1 (2.2–4.3)
60–90	119	2.4 (1.7–3.3)
Decompensated cirrhosis		
−90 to −60	196	3.1 (2.4–4.1)
−59 to −30	211	3.4 (2.5–4.4)
−29 to −1	239	4.2 (3.1–5.4)
0–29	664	9.4 (7.5–11.8)
30–59	225	5.8 (4.0–8.4)
60–90	142	3.3 (2.4–4.7)
By compensated cirrhosis etiology		
Alcohol-associated cirrhosis		
−90 to −60	127	4.1 (2.9–5.9)
−59 to −30	105	1.9 (1.4–2.6)
−29 to −1	546	8.5 (6.5–11.0)
0–29	223	6.5 (4.6–9.2)
30–59	74	2.8 (1.8–4.3)
60–90	69	3.0 (1.8–4.8)
Cirrhosis due to other etiologies		
−90 to −60	77	3.6 (2.3–5.6)
−59 to −30	102	6.4 (3.9–10.4)
−29 to −1	366	15.3 (10.3–23.0)
0–29	139	9.5 (5.7–15.8)
30–59	55	3.4 (2.0–5.8)
60–90	50	1.9 (1.2–3.0)

^a^ *p*<0.001 for all IRRs in the table.

**FIGURE 2:**
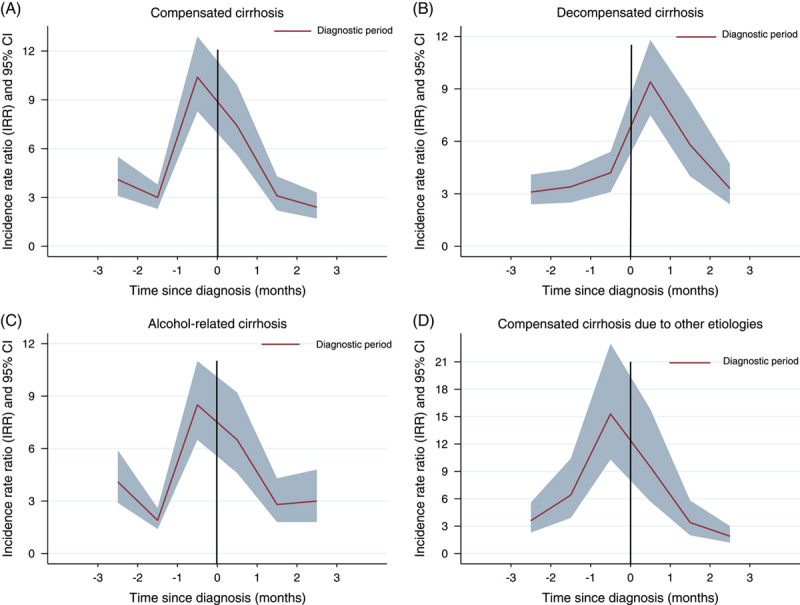
Incidence rate ratio of injuries during diagnostic period compared to prediagnostic period in compensated cirrhosis (A), decompensated cirrhosis (B), alcohol-associated cirrhosis (C), and compensated cirrhosis due to other etiologies (D).

In the subgroup analysis by cirrhosis severity and etiology, the risk of injuries started to increase 2 months before and was highest during 1 month before the diagnosis among patients with compensated cirrhosis both due to alcohol and other causes(Table 3 and Figure 2). The risk then declined constantly over time after the diagnosis date. On the contrary, for patients with decompensated cirrhosis, the increased risk of injuries peaked 1 month after cirrhosis diagnosis and remained relatively high also thereafter.

### Injury subtypes

For the full cohort, we observed an increased risk of falls (IRR 9.9, 95% CI 9.1–11.0) and fractures (IRR 7.4, 95% CI 6.7–8.3) during the diagnostic period than the prediagnostic period (Table 4). Among patients with fractures, the risk increase was mainly attributed to osteoporotic fractures (IRR 8.6, 95% CI 7.4–9.9) and fractures of the hip or pelvis (IRR 12.9, 95% CI 10.5–15.8) and the skull, vertebrae, ribs, or sternum (IRR 9.7, 95% CI 7.6–12.0). We did not observe an increased risk of self-harm during the diagnostic period.

**TABLE 4:** Incidence rates (IRs) per 1000 person-months and incidence rate ratios (IRRs) of injuries by injury subtypes in patients with cirrhosis

	Prediagnostic period		Diagnostic period			
	No. injuries	IR	No. injuries	IR	IRR	*p*
Falls	568	4.0	2459	7.9	9.9 (9.1–11.0)	<0.001
Fractures	511	3.6	1776	5.7	7.4 (6.7–8.3)	<0.001
By location						
Skull/vertebrae/ribs/sternum	110	0.8	485	1.6	9.7 (7.6–12.0)	<0.001
Hip/pelvis	133	0.9	692	2.2	12.9 (10.5–15.8)	<0.001
Extremities	273	1.9	631	2.0	4.2 (3.6–4.9)	<0.001
By mechanism						
Osteoporotic fractures	283	2.0	1079	3.5	8.6 (7.4–9.9)	<0.001
High-energy fractures	66	0.5	157	0.5	4.2 (3.1–5.8)	<0.001
Others	181	1.3	613	1.9	7.2 (5.9–8.5)	<0.001
Self-harm	27	0.1	35	0.1	1.5 (0.8–2.6)	0.130

### Sensitivity analyses

We found that the higher rate of injuries among patients with cirrhosis could not be explained by increasing age, after comparing the incidence rate in patients with cirrhosis to that of matched controls from the general population of the same age (Supplemental Figure S1, http://links.lww.com/HC9/A561).

We identified 17,713 (29.8%) deaths among patients with cirrhosis, of which 417 could be attributed to injuries during the diagnostic period. We found similar estimates for compensated cirrhosis and slightly higher estimates for decompensation when we restricted the analysis to patients who were alive throughout the study period (Supplemental Table S3, http://links.lww.com/HC9/A561), indicating that censoring for death did not significantly impact the studied association.

## DISCUSSION

In this study including more than 50,000 patients with cirrhosis in Sweden, we observed that injuries were common just before a diagnosis of cirrhosis, suggesting that cirrhosis is often diagnosed in close conjunction with an injury that requires health care. The higher rate of injuries compared to the matched population controls suggests an association between the consequences of subclinical cirrhosis and a higher risk of injuries. For patients with compensated cirrhosis, the peak increase in risk was shortly before cirrhosis diagnosis, in contrast to patients with decompensation where the risk increase was highest after cirrhosis diagnosis. Falls and fractures were the most common injury types, and this finding indicates that better identification of modifiable risk factors is of high importance. To the best of our knowledge, this is the first assessment of the injury risk both before and after a diagnosis of cirrhosis, using a self-comparison design that expands our understanding of the clinical course of cirrhosis with better controlling of confounding factors.

### A diagnosis of cirrhosis heralds vulnerability to falls and fractures

Our data confirm prior work demonstrating a high risk of fractures after a diagnosis of cirrhosis,^9,10^ and extend the literature by highlighting the risks during the diagnostic period. First, we demonstrate that patients with cirrhosis incur injurious falls at a rate of 4.1% during 6 months. This is less frequent than prior estimates from prospectively followed patients (where the probability of injurious falls was 9.1% at 1 y), likely because our study relied on patients presenting for care to the hospital.^24^ Second, our self-controlled case series design allows the comparison of different periods within individuals and across the clinical course of cirrhosis. Owing to the asymptomatic nature of compensated cirrhosis, a spike of injury risk before the diagnosis might result from an opportunistic detection, in that injury event precipitated the patients to hospital admission, at which point the cirrhosis could be identified. There might be several reasons why injuries are linked to incident cirrhosis diagnoses. Patients with compensated cirrhosis often have underlying hepatic osteodystrophy and are prone to falls and fractures,^25^ and minimal HE influences complex cognitive or coordination skills such as driving leading to an increased risk of accidents.^26^ Especially in patients with alcohol-associated cirrhosis, the risk of injuries may be further increased by the direct effect of alcohol (eg, disorientation) or its sequelae including malnutrition and neuropathy.^27^ On the other hand, a higher risk of injuries was noted also for compensated cirrhosis due to other etiologies where other factors may account for the presence of injury.^28,29^ For example, patients with cholestatic liver disease might have osteoporosis due to low uptake of fat-soluble vitamin D,^30^ and patients with NAFLD might be prone to falls and fractures due to insulin resistance-related factors.^31^

Third, patients with decompensation had a consistently increased risk of injuries and the risk increment was highest 1 month after the diagnosis. More severe sarcopenia and cognitive disturbance might already present before decompensated cirrhosis is diagnosed, which are likely to set the stage for subsequent injuries in these patients. These conditions might become more pronounced at the time of decompensation, explaining the high risk of injuries that was noted thereafter. Our results are in concordance with the earlier finding that the highest risk of fractures was observed immediately after a diagnosis of decompensated cirrhosis.^9^ An increase in rate of injuries immediately after decompensated cirrhosis can also be worsened by polypharmacy such as nonselective beta-blockers or diuretics during admission, or by orthostatic hypotension. Apart from the long-term prevention of injuries, strategies targeting the time window shortly after cirrhosis are justified to decrease the risk of injuries.

### Injury subtypes

We found that hip fractures seem to be the predominant injury, and osteoporosis is the main presumed mechanism leading to fractures, in line with studies.^8,10^ In addition, we did not observe a significant association between the diagnosis of cirrhosis and the risk of self-harm, in contrast to 2 other studies of a higher risk of suicidal attempts among patients after receiving the diagnosis of cirrhosis.^6,32^ The limited number of self-harm cases in our study may hamper a meaningful interpretation of the results.

### Strengths and limitations

The strengths of this study include a large number of patients with cirrhosis, a previously confirmed high PPV of the register-based diagnosis of cirrhosis and almost complete follow-up due to the high-quality national registers.^20,33^ The use of self-controlled case series design limits the potential impact of unmeasured confounders, assuming that these confounders are constant within individuals over a short period of time. However, this design is sensitive to time-varying factors, such as increasing age and progression to a major disease. The impact of increasing age on injuries was partly ruled out in our study, as indicated from the sensitivity analysis. However, among patients with cirrhosis, the increasing age might still affect the occurrence of injuries, especially for the elderly who are more vulnerable to injuries. Other time-varying risk factors, such as initiation of injury-related drugs (eg, benzodiazepines) or development of other disease, might affect injury ascertainment during the cirrhosis diagnostic period. In addition, we could use record data from secondary and tertiary care, since the NPR does not capture diagnoses made in primary care. Injuries included in our study might represent acute injuries such as fractures that referred to an emergency room or more severe cases. Although the PPVs for diagnoses used in this study are high,^20^ patients with undiagnosed cirrhosis could not be identified. However, most patients with both injuries and decompensated cirrhosis are in contact with specialized health care, meaning they would be identified by our algorithm.

### Clinical implications

It is difficult to diagnose compensated cirrhosis due to its covert nature, and our study shows that 5.6% of all patients with newly diagnosed compensated cirrhosis had their diagnosis made in conjunction with an injury, suggesting that this injury event led to *en passant* discovery of cirrhosis. Early detection of cirrhosis remains a challenge, and with the incidence and mortality rates rising, the motive of investing in prevention and early detection of cirrhosis has never been stronger. Our findings have significant implications for clinicians who work in first line with patients with an injury and could be of at higher risk for cirrhosis, for example, with a history of alcohol-associated disorder. The presence of an injury could be indicative of cirrhosis, thus warranting the consideration of cirrhosis screening for such patients. Our result of a high risk of injuries shortly after the diagnosis of cirrhosis provides a critical time window for injury prevention, especially relevant for patients with decompensated cirrhosis. As a group at high risk of falls and fractures, patients with decompensation would benefit from multiple interventions aiming at reducing their risks. This includes counseling regarding alcohol consumption and nutritional support, intensifying physical training including leg strength and balance exercises such as Tai Chi, reducing polypharmacy (in particular sedating medications),^34^ and initiating HE-directed therapy for those with covert HE.^28^ Further research is needed to identify the specific causes of injuries and any prevention strategies needed to reduce the risk, for example, postdischarge falls and fractures.

In this large study of patients with cirrhosis, we show that the risk of injuries is high shortly before, and after, a diagnosis of cirrhosis and provide risk estimates for such outcomes. Our results call for targeted prevention of falls and fractures after a diagnosis of cirrhosis.

## Supplementary Material
