# Supplementary material for: Risk of injuries before and after a diagnosis of cirrhosis: A population-based cohort study
Source: Hepatol Commun. 2023 Oct 12;7(11):e0238. doi: 10.1097/HC9.0000000000000238 (PMC10578726; doi:10.1097/HC9.0000000000000238)
Supplement: Supplementary file 1 [file hc9-7-e0238-s001.docx]

**Risk of injuries before and after a diagnosis of cirrhosis: a population-based cohort study**

Ying Shang, Qing Shen, Elliot B. Tapper, Axel Wester, Hannes Hagström

**Appendix: Supplement material**

**eFigure 1-** Incidence rate of injuries preceding the cirrhosis diagnosis per 1,000 person-months for patients with cirrhosis and individuals from the general population matched for age, sex, and municipality.

**eTable 1-** ICD codes for cirrhosis, injuries, and comorbidities

**eTable 2-** Number and incidence rate of injuries preceding the cirrhosis diagnosis per 1,000 person-months for patients with cirrhosis and individuals from the general population matched for age, sex, and municipality.

**eTable 3-** Incidence rate ratio of injuries during cirrhosis diagnosis with cases who died during the observation period excluded

**
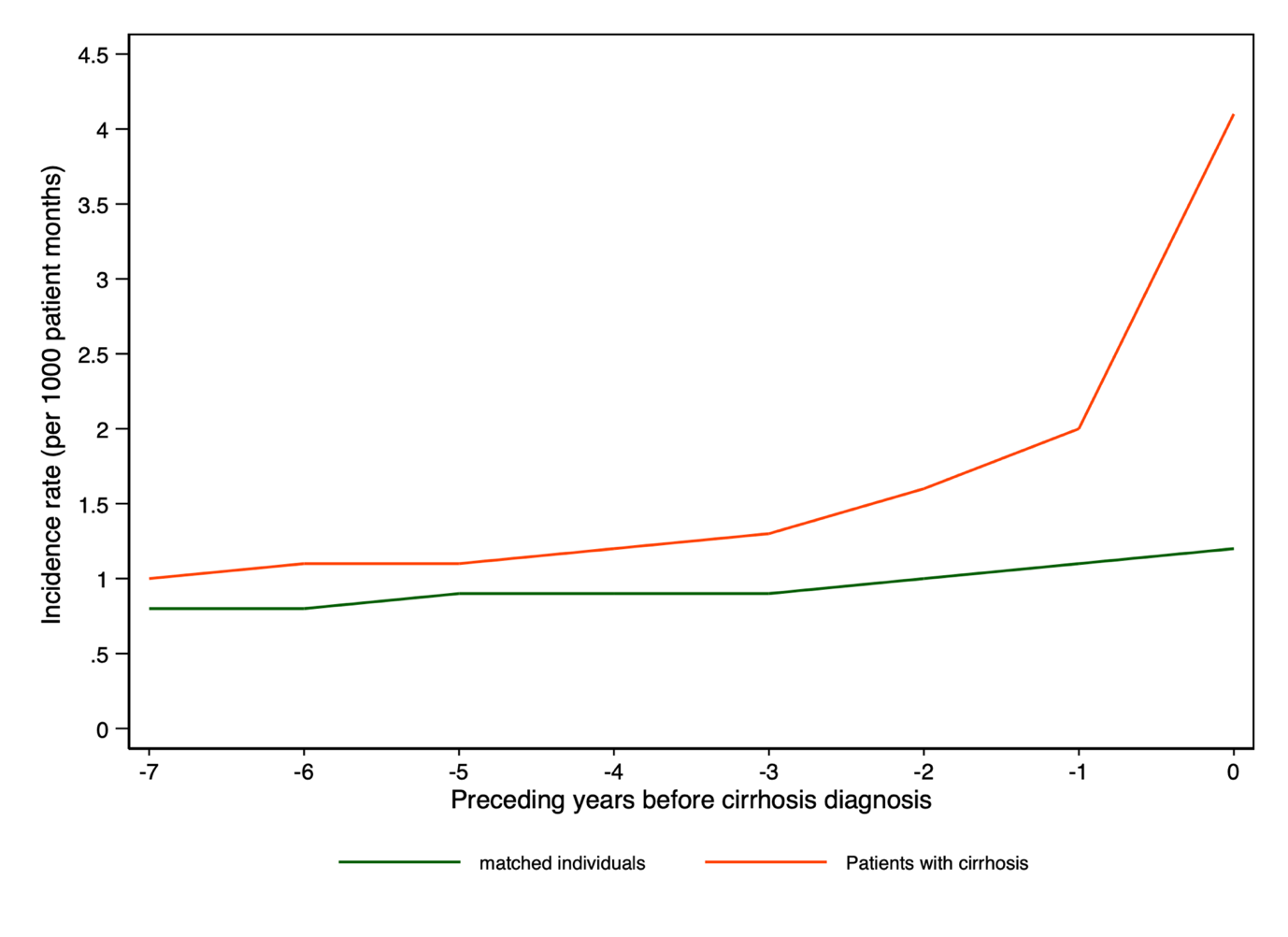
eFigure 1**. Incidence rate of injuries preceding the cirrhosis diagnosis per 1,000 person-months for patients with cirrhosis and individuals from the general population matched for age, sex, and municipality.

**eTable 1**. ICD codes for cirrhosis, injuries, and comorbidities

**Table A**. ICD codes for cirrhosis

Cirrhosis was identified through hospital discharge records from the Swedish Patient Register using the diagnosis coded according to the 10th (1997-2019) Swedish revision of the International Classification of Diseases (ICD)

| **Diagnosis** | **ICD-10** |
| --- | --- |
| **Cirrhosis** |  |
| Alcohol-related cirrhosis | K70.3 |
| Cirrhosis due to other etiologies | K74.6, B18.2G, B18.2E, B18.1G, B18.1E, B18.0G, B18.0E, B18.8G, B18.8E |
| **Decompensated Cirrhosis** |  |
| Ascites | R18.9 excluding cancer and congestive heart failure within 1 year |
| Esophageal varices with bleeding | I85.0, I98.3 |
| Esophageal varices without bleeding | I85.9, I98.2 |
| Hepatic encephalopathy | Any compensated cirrhosis code+rifaximin *(A07AA11)/lactulose*(A06AD11) |
| Hepatorenal syndrome | K76.7 |
| Chronic liver failure | K72.1 |
| Liver failure | K72.9 |
| Portal hypertension | K76.6 |

*Treatment was obtained based on Anatomical Therapeutic Chemical classification from the Prescribe Drug Register

**Table B**. ICD codes for injuries

All injuries were identified from hospital discharge records from the Swedish Patient Register using the diagnosis coded according to the 9th (1987-1996) and 10th (1997-2020) Swedish revisions of the International Classification of Diseases (ICD) codes together with E-codes which indicate the external causes of injury.

| **Diagnosis** | **ICD-10** | | **ICD-9** | |
| --- | --- | --- | --- | --- |
|  |  | E-code |  | E-code |
| **All Injuries** | S00-S99, T00-T35, T51-T79, T89-T95, T97, T98.0-T98.2 |  | 800-959, 980-989, 990-995, 9091, 9092, 9094, 9096-9099 |  |
| Falls |  | Any injury +W00-W19, X80, Y01, Y30 |  | Any injury +E880-E886, E888, E957, E968.1, E987 |
| Fractures | S02, S12,S22,S32,S42,S52,S62,S72,S82,S92  T02,T08,T10,T12,M80,M485,M495 |  | 733.1  800-829 |  |
| By mechanisms |  |  |  |  |
| Osteoporotic fractures | S12, S22.0, S22.1, S32.0-S32.2. S32.3-S32.7, S42.2, S52.5, S52.6, S72.0-S72.2 M80, M48.5, M49.5 |  | 805-806, 808, 812, 813, 820 |  |
| High-energy fractures |  | Any fracture+V02-09, V11-79, W10-17 |  | Any fracture+E807/819/880-884 |
| By location |  |  |  |  |
| Skull/Vertebrae/Ribs/Sternum | S02, S12, S22.0, S22.1, S32.0-S32.2, S32.7, M48.5, M49.5, S22.2-S22.5, S22.8, S22.9 |  | 800-807 |  |
| Hip/Pelvis | S72.0-S72.2, S32.3-S32.5, S32.7 |  | 808, 820 |  |
| Extremities | S42.0-S42.4, S42.7-S42.9, S52, S62, S72.3, S72.4, S72.7-S72.9, S82, S92 |  | 810-817, 821-826 |  |
| Self-harm |  | Any injury+X60-X84, Y870 |  | Any injury+E950-E959 |

**Table C**. ICD codes for comorbidities

All comorbidities were identified from hospital discharge records from the Swedish Patient Register using the diagnosis coded according to the 9th (1987-1996) or 10th (1997-2020) Swedish revisions of the International Classification of Diseases (ICD)

| **Diagnosis** | **ICD-10** | **ICD-9** |
| --- | --- | --- |
| Cardiovascular disease | I20-I24, I61, I63, I64, I110, I130, I132, I255, I420, I426, I427, I428, I429, I43, I50 | 410-413,431, 433, 402A 402B 402X 404A 404B 404X 425E 425F 425H 425W 425X 428 |
| Congestive heart failure | I110, I130, I132, I255, I420, I426, I427, I428, I429, I43, I50 | 402A 402B 402X 404A 404B 404X 425E 425F 425H 425W 425X 428 |
| Cancer except hepatocellular carcinoma | C00-C21, C23-C97，  D00-09, D10-36, D37-48 | 140-154, 156-199, 210-239 |
| Psychological disorder | F1-F9 | 291-319 |
| Dementia | F00- F03, F051, F107, G30 | 290, 294B, 331A-C |
| Diabetes | E10-E14 | 250A-250G |
| Osteoprosis | M80-M82 | 733A |

**eTable 2**. Number and incidence rate (IR) of injuries preceding the cirrhosis diagnosis per 1,000 person-months for patients with cirrhosis and individuals from the general population matched for age, sex, and municipality.

|  |  | -7 years | -6 years | -5 years | -4 years | -3 years | -2 years | -1 year | 0 year |
| --- | --- | --- | --- | --- | --- | --- | --- | --- | --- |
| Patients with cirrhosis | n | 710 | 789 | 817 | 888 | 967 | 1165 | 1418 | 2901 |
|  | IR | 1.0 | 1.1 | 1.1 | 1.2 | 1.3 | 1.6 | 2.0 | 4.1 |
| Individuals from the general population matched for age, sex, and municipality | n | 5475 | 5740 | 6072 | 6132 | 6257 | 6831 | 7272 | 7795 |
|  | IR | 0.8 | 0.8 | 0.9 | 0.9 | 0.9 | 1.0 | 1.1 | 1.2 |

**eTable 3.** Incidence rate ratio (IRR) of injuries during cirrhosis diagnosis after excluding cases who died during the observation period

|  | Pre-diagnostic period | | Diagnostic period | | |  |
| --- | --- | --- | --- | --- | --- | --- |
|  | n. injuries | IR | n. injuries | IR | IRR (95% CI) | *P* value |
| All cirrhosis | 707 | 2.8 | 2,618 | 10.8 | 7.8 (7.1-8.5) | < .001 |
| Compensated cirrhosis | 367 | 3.2 | 1,544 | 14.1 | 9.6 (8.5-10.8) | < .001 |
| Alcohol-related cirrhosis | 245 | 4.2 | 965 | 17.2 | 8.8 (7.5-10.3) | < .001 |
| Compensated cirrhosis due to other etiologies | 122 | 2.2 | 579 | 10.9 | 11.1 (8.9-13.7) | < .001 |
| Decompensated cirrhosis | 340 | 2.5 | 1,074 | 8.1 | 5.9 (5.1-6.7) | < .001 |
| Bleeding esophageal varices | 46 | 2.3 | 161 | 8.4 | 7.0 (4.9-10.1) | < .001 |
| Ascites | 248 | 2.3 | 799 | 7.7 | 6.1 (5.2-7.1) | < .001 |
